# Body representation drives auditory spatial perception

**DOI:** 10.1016/j.isci.2024.109196

**Published:** 2024-02-12

**Authors:** Daniel Paromov, Karina Moïn-Darbari, Assan Mary Cedras, Maxime Maheu, Benoit-Antoine Bacon, François Champoux

**Affiliations:** 1 Université de Montréal, Montréal, QC, Canada; 2 Centre de recherche de l’Institut Universitaire de Gériatrie de Montréal, Montréal, QC, Canada; 3 Department of Psychology, The University of British Columbia, Vancouver, BC, Canada

**Keywords:** Health sciences, Human activity in medical context, Social sciences

## Abstract

In contrast to the large body of findings confirming the influence of auditory cues on body perception and movement-related activity, the influence of body representation on spatial hearing remains essentially unexplored. Here, we use a disorientation task to assess whether a change in the body’s orientation in space could lead to an illusory shift in the localization of a sound source. While most of the participants were initially able to locate the sound source with great precision, they all made substantial errors in judging the position of the same sound source following the body orientation–altering task. These results demonstrate that a change in body orientation can have a significant impact on the auditory processes underlying sound localization. The illusory errors not only confirm the strong connection between the auditory system and the representation of the body in space but also raise questions about the importance of hearing in determining spatial position.

## Highlights

• Change in body orientation leads to illusory shifts in sound localization
• Auditory cues alone are not sufficient to maintain orientation of the body in space
• Auditory spatial perception is dominated by body representation in space

## Graphical abstract

**Figure:**
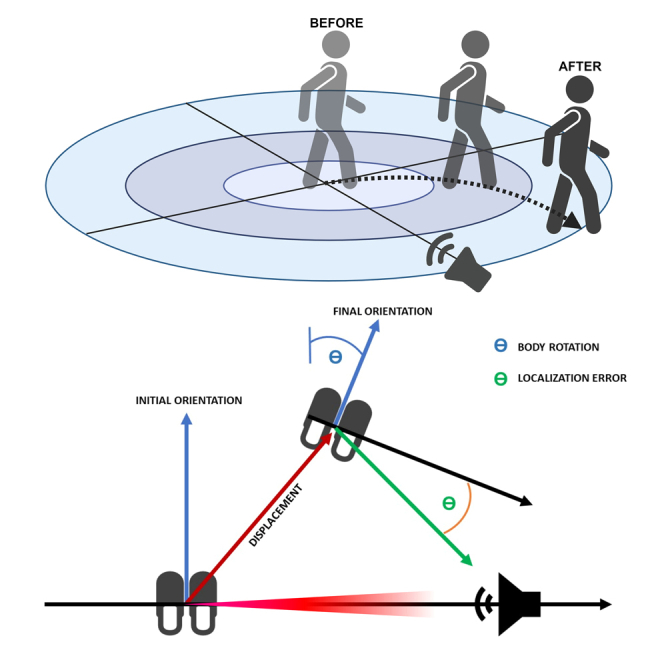

## Introduction

The representation of the body in space is a complex process that requires the joint integration of several sensory modalities, namely visual, somatosensory, and vestibular.^1^ For more than a decade now, the contribution of auditory function to this process has been increasingly investigated. Indeed, several studies suggest a significant role of auditory input in processes related to body perception.^2^ For example, research reveals that auditory stimuli can improve postural control in normal individuals.^3^ In addition, moving auditory stimuli congruent with body movements can help position the body in space.^4^ Conversely, incongruent auditory stimuli can induce vection or the sensation of illusory self-movement.^5^^,^^6^^,^^7^^,^^8^

While the effects of auditory stimulation on postural control and balance have been clearly demonstrated, the contribution of the auditory system in the representation of the body in space is not well understood. Some researchers suggest that auditory input, through the localization of auditory elements in the environment, could provide sufficient information to position the body in space relative to its environment.^9^^,^^10^^,^^11^^,^^12^ Related to this hypothesis, it has been more specifically proposed that the auditory system could allow for the stabilization of the body during vestibular disturbances throughout this spatial localization.^12^

The inverse interaction, namely the influence of body representation on auditory processes, remains essentially unexplored. Sound localization is the result of a computational process based on binaural signals. This process, in the broadest sense, results from the integration of information from different senses.^13^ For sounds to be localized, they must be positioned in space relative to the body. This suggests an important reciprocal association between the auditory system and other sensory elements allowing representation of the body in space.

In line with such assumptions, several experiments have demonstrated the influence of sensorimotor inputs related to the body (i.e., motor, tactile, vestibular) on auditory perception.^14^^,^^15^^,^^16^^,^^17^^,^^18^^,^^19^^,^^20^^,^^21^^,^^22^^,^^23^^,^^24^^,^^25^ Although the impact of the motor and sensory domains on the auditory processes are significant, their influences remain negligible, suggesting an excellent stability in the neural processing of auditory spatial cues in humans.^14^

To date, the influence of a disturbance in the representation of the body on the auditory processes has never been explored. Therefore, it is unclear whether a disruption of this representation, which is constructed from all the various aforementioned sensorimotor elements, has a noticeable and measurable impact on auditory perception. In the present study, we used a classic disorientation task to examine whether a change in the representation of the body in space could lead to a significant illusory shift in the localization of a sound source. Indeed, participants were unable to localize a sound source with precision while performing the Fukuda-Unterberger stepping test.^26^ The illusory shift that resulted suggests that auditory spatial perception is entirely dominated by body representation in space.

### Methods

Twenty participants (mean age = 23.8; SD = 1.6; 16 women and 4 men) with normal auditory function (mean group pure tone average for 500, 1000, 2000, and 4000 Hz of 0.5 dB HL; SD = 4.9 for the left ear and 0.8 dB HL, SD = 5.4 for the right ear; no auditory thresholds above 15 dB HL), no history of vertigo, and normal vestibular function, as assessed by video head impulse test (see Nooristani et al., for detailed examination procedure^27^) were included in the data analysis. All participants presented with the same educational level background and reported no known motor or cognitive disability which would impact performance. All participants reported normal or corrected-to-normal vision. Participants did not have a history of diabetes, head trauma, cervical disorders or neurological disorders.

Participants were asked to remain in the same location while performing the Fukuda-Unterberger stepping test,^26^ a task known to disrupt spatial orientation of the body in space. Participants were blindfolded throughout the experiment and had no previous visual knowledge of the experimental layout of the room. They stood in the middle of a quiet room with their arms outstretched directly in front of them. They were asked to step in place, without moving forward, at a rate of 2 steps per second for 60 s. During the task, a rotation of the body in the horizontal plane was observed in all participants in at least one auditory stimulus positions, without them being aware of the change in body orientation. No exclusions were made on the basis of the disturbance effect of the task performed.

Participants performed the task while a speech sequence consisting of a male voice narrating a series of sentences with minimal pauses, at 55 dB SPL, in a language well understood by all participants (English). The speech sequence was presented at 0, 45 or 90° azimuth at the level of the participant’s head through a speaker (Sound Source Type 4224, Bruel & Kjaer, Denmark) positioned at a distance of 2 m. Each position was tested 3 times in a pseudorandom order. This stimulation sequence was chosen based on a pilot study carried out in 21 participants. The results of the pilot study revealed that this sequence was the only one generating very low localization variability after a duration of 60 s (i.e., below 8 degrees of error in localization).

During the experimental task, participants were told that the sound source was fixed and they were asked to use the sound to maintain their position, i.e., keep the sound source at the same distance and at the same degrees azimuth to their head. Without moving their head, they were asked to locate the sound before (static condition) and after performing the 60-s task by using a laser pointer placed in their right hand, which allowed accurate measurements of the deviation from the sound source. The magnitude of the errors in localization (perceived position – real position), the traveled distance, and the rotation of the body relative to the initial 0° straight ahead were collected and measured by the experimenter using a goniometer, which allows for the detection of a change in position with a precision of 1°. A paired sample t-test was performed to examine the differences in body position in each individual before and after the task.

## Results

**Figure 1:**
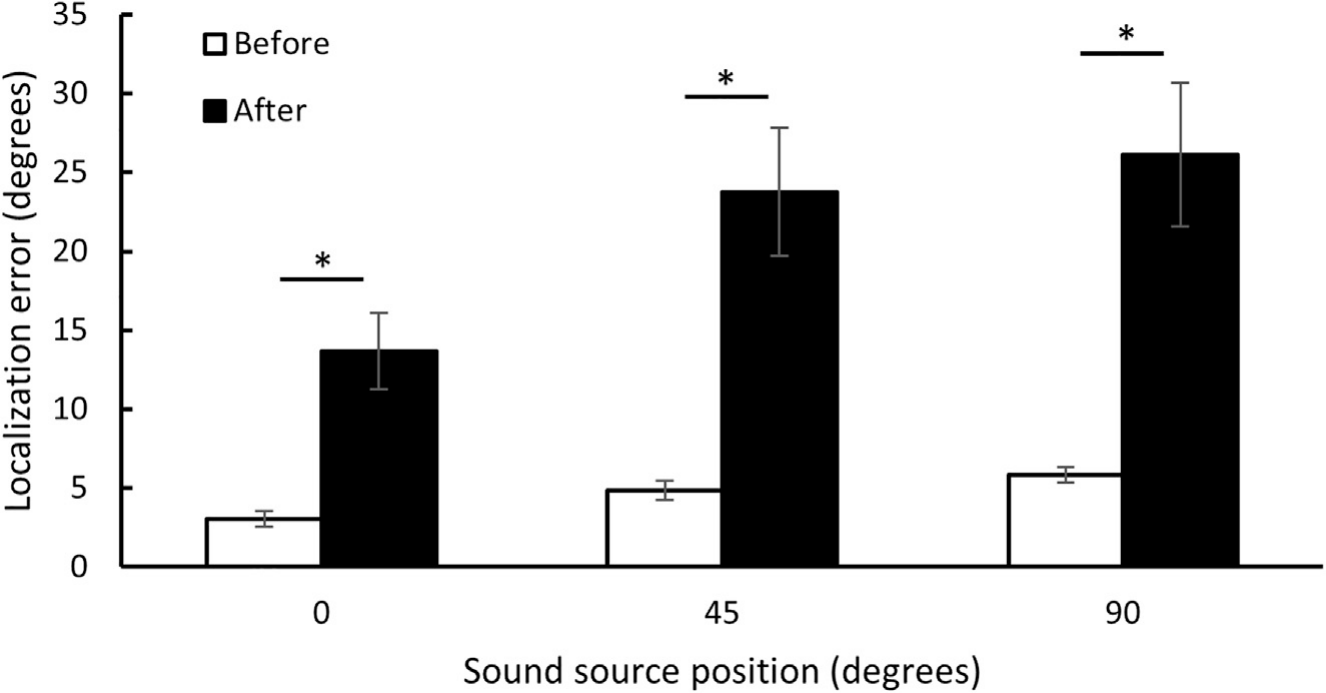
Boxplot showing the magnitude of sound localization errors before and after the 60-s stepping task The auditory stimulation is presented at 0, 45, and 90° and does not vary in position during the task. Before the disorientation task, the participants are able to locate the sound source with precision at 0 (mean = 3, SE = 0.49), 45 (mean = 5, SE = 0.6) and 90° (mean = 6, SE = 0.5). Following the disorientation task, the participant is likely to make a significant error in locating the sound source at 0 (mean = 13, SE = 2.4), 45 (mean = 24°, SE = 4.1), and 90° (mean = 26, SE = 4.6). ∗p < 0. 001.

Before the task, all participants were able to locate the sound source with precision at 0° (mean error = 3, SD = 2.2), 45° (mean error = 5, SD = 2.6), and 90° (mean error = 6, SD = 2.2), but made significant errors after the perturbation task with the sound at 0 (mean error = 13, S = 10.8), 45 (mean error = 24, SD = 8.2), and 90° (mean error = 26, SD = 20.4). Paired comparisons revealed no differences between repetitions one, two, and three for all the sound source positions, both before and after the perturbation task (p > 0.05). Body rotations, ranging up to 77°, were observed following the stepping task, suggesting the presence of an illusory shift in sound position at all positions (see Figure 1).

Repeated measures ANOVAs were conducted for each sound source position (0,45,90) × 2 measurement times (before, after). There was a significant effect of measurement time [F(1,19) = 28.604. p < 0.001, η^2^ = 0.601] and sound source position [F(2,38) = 8.211, p = 0 0.001, η^2^ = 0.428 ]. There was also a significant interaction between measurement times and sound source positions [F(2,38) = 4.210, p = 0.022, η^2^ = 0.322]. Paired comparisons using Bonferroni correction revealed that errors in localization were significantly greater after perturbation at all sound source locations (0°: [t(19) = −4.402, p < 0.001], 45°: [t(19) = −4.718, p < 0.001], 90°: [t(19) = −4.542, p < 0.001]. Sound source position had a relative impact on the magnitude of the shift in localization, as errors were significantly smaller at 0° compared to 45° [t(19) = −2.746, p = 0.13] and 90° [t(19) = −3.217, p = 0.005]. Results suggest that errors in localization were similar when presented more laterally, namely at 45° and 90° [t(19) = −0.651, p = 0.523]. As expected, errors in localization were correlated with body rotation (0°: [r(18) = 0.667, p = 0.001], 45°: [r(18) = 0.630, p = 0.003], 90°:[r(18) = −0.504, p = 0.024]). Localization errors were not correlated with traveled distance, age or hearing acuity (p > 0.05).

At the end of the experiment, all participants testified that they were completely unaware of a change in body orientation and felt confident of the orientation of their body in relation to the sound source during the entire testing session.

## Discussion

In the present study, we aimed to examine whether a change in the orientation of the body in space, without awareness that body orientation was altered, could lead to a significant illusory shift in the localization of a sound source. The results clearly demonstrate that a change in the representation of the body in space could have a significant impact on the auditory processes underlying sound localization and therefore confirm that sound localization is a complex multisensory process.

Sound localization is a computational process based on binaural and spectral cues,^28^ but the well-known psychoacoustic rules do not apply when the auditory system interacts with incongruent sensory cues related to body representation. Indeed, auditory signals require clear spatial referencing. To determine its position in space, the brain integrates information from multiple sensory modalities and, through this process, generates a coherent and apparently seamless percept of the external world.^29^ Although multisensory integration typically binds information that is derived from the same event, when multisensory cues are somewhat discordant, they can result in illusory percepts (for a review in the audiovisual domain, see Opoku-Baah et al.).^30^ Such interaction is the result of the integration of sensory information in structures such as the superior colliculus, a mid-brain structure known for its involvement in sensorimotor integration and in orienting behavior in 3D space.^31^^,^^32^ If the results confirm a strong interaction between the auditory system and sensorimotor input related to the body, the neural substrates of these interactions might be difficult to determine, especially considering the complex and dynamic processing that underpins body representation.

The reported interaction no doubt emerges in the early stages of life and is shaped by experience. Indeed, the role of auditory input in the emergence and development of body-related processes has recently been demonstrated by Ronga et al.^33^ The study shows a primitive coding of body boundaries in newborns based on the spatial tuning of electrophysiological responses to multisensory stimuli. Remarkably, they also show that audio-tactile integration is already present and spatially organized in 18–92-hour-old newborns. The role of audition in body-related processes is also supported by research showing that hearing loss can lead to impairments in several postural and motor tasks (for a review, see Houde et al.).^2^ The present results add to this body of work by confirming the strong connection between both sensory systems.

Nonetheless, the results suggest that the interaction is somewhat unbalanced. In the current experiment, participants were initially able to locate the sound source with great precision, and the accurate positioning of the sound source could have been used as a reference to stabilize the body. However, the results suggest that auditory cues were not sufficient to adequately maintain orientation of the body in space in the absence of visual cues. Future studies should aim to develop other spatial disorientation tasks in order to more accurately pinpoint the parameter triggering this auditory shift in perception.

Data on the functions and processing carried out by the auditory system to perceive sounds are becoming increasingly precise. On the other hand, the complex reciprocal interactions between auditory and other sensory modalities as they pertain to orienting the body in space remains to be assessed in a more detailed manner. It is essential to better understand the sensory elements that allow for an adequate representation of the body in space in order to understand some of the perceptual problems linked to this process. Indeed, beyond improving our understanding of the fundamental basis of sound localization, which could definitely have implications on the understanding of auditory difficulties (e.g., auditory scene analysis deficits, auditory hallucinations), the data could lead to a better grasp of multiple issues related to body representation in space (e.g., motion sickness, cybersickness, space adaptation syndrome) and could, therefore, have significant implications for the further development of technologies such as virtual reality and telepresence.

### Limitations of the study

The stimulation parameters underlying performance are not completely understood. Indeed, although the illusory shift in sound source localization was found at all spatial positions examined, our results suggest that sounds presented laterally lead to a more significant illusory shift compared to sounds presented in front of the participants. This suggests that certain spatial orientations or positions are more resistant or susceptible to changes in body position. Further investigation is needed to isolate the stimulation parameters leading to these results, and to better understand the individual differences that might influence the interaction. This would help to better define the relative importance of the auditory system in the determination of body orientation in space.

## STAR★Methods

### Key resources table

**Table:** 

REAGENT or RESOURCE	SOURCE	IDENTIFIER
**Deposited data**		

Raw data	This paper	Open Science Framework: https://doi.org/10.17605/OSF.IO/ANCDR

**Software and algorithms**		

G∗Power 3.1.	Faul et al.^34^	https://www.psychologie.hhu.de/arbeitsgruppen/allgemeine-psychologie-und-arbeitspsychologie/gpower

### Resource availability

#### Lead contact

Further information and requests for resources and reagents should be directed to and will be fulfilled by the lead contact, François Champoux (Francois.champoux@umontreal.ca).

#### Materials availability

This study did not generate new unique reagents.

#### Data and code availability

• Data have been deposited on the Open Science Framework: https://doi.org/10.17605/OSF.IO/ANCDR and are publicly available as of the date of publication.
• This paper does not report original code.
• Any additional information required to reanalyze the data reported in this work paper is available from the lead contact upon request.

### Experimental model and subject details

#### Human participants

The experiment was performed by 20 participants. Accordingly, the data of 20 participants were analyzed (mean age=23.8; SD=1.6; 16 women and 4 men). Race and ancestry were not considered in the study design as there was no evidence that the perturbation task (Fukuda) or auditory task could be biased by these parameters.

All participants had normal hearing and vestibular function. All participants gave informed consent before participating. The experiment was approved by the University of Montreal ethics committee.

#### Sample size quantification

The sample sizes for these experiments were chosen in advance. The sample size is based on a repeated measures ANOVA power calculation (one group, 3 repeated measures, effect size 0.4, significance level .05, power .95) using G∗Power 3.1.^34^

### Method details

The experiment was performed in a dimly lit room. Participants were blindfolded prior to entering the room and were guided to a specified location marked on the floor. Participants received a laser pointer which was placed in their right hand.

The stimuli were presented through a speaker (Sound Source Type 4224, Bruel & Kjaer, Denmark) and consisted of a speech sequence consisting of a male voice narrating a series of sentences with minimal pauses, at 55 dB SPL at a distance of 2 m. Participants were asked to point to the location of the sound source (measure 1: Difference between perceived position and real position). After performing the task consisting of a static military march with the hands extended in front of the participants (Fukuda, 1959), they were again asked to locate the sound source (i.e., point at exactly 0; 45; 90 degrees azimuth, as per the task instruction that was given before conducting the experiment which stated to keep the sound source at exactly the same position during the task), at this point the difference between the real position of the sound source and the perceived position by the participants was measured using a goniometer (measure 2). Before the participants left the experimental room, their final position and orientation were marked on the floor for further measurements. The mark was subsequently used to determine the distance traveled from the initial position, and the rotation of the body relative to the initial 0-degree position.

### Quantification and statistical analysis

Error in localization was computed as the difference between the real position of the sound source – and the perceived position of the sound source. To test for differences in the perceived position of the sound source, a repeated measures 3×2 ANOVA was carried out using both variables. Post-Hoc analysis for all of the follow-up tests were carried out using the Bonferroni correction. Subsequent Pearson correlations were used to reveal any effect of age, sex, hearing acuity, distance traveled, and body rotation.
